# Is Tranexamic Acid Safe and Efficacious in Hip Surgeries?

**DOI:** 10.7759/cureus.21249

**Published:** 2022-01-14

**Authors:** Karthik SJ, Prabhu Ethiraj, Arun H Shanthappa, Kishore Vellingiri

**Affiliations:** 1 Department of Orthopedics, Sri Devaraj Urs Medical College, Sri Devaraj Urs Academy of Higher Education and Research, Kolar, IND

**Keywords:** deep vein thrombosis, blood transfusion, blood loss, hip surgeries, tranexamic acid

## Abstract

Background

The incidence of hip fractures is increasing in the current population. It is estimated by the year 2050 around 6.3 million hip fractures may occur per year. Management of hip fractures and replacement surgeries might be associated with substantial blood loss which leads to perioperative anemia. Tranexamic acid is an antifibrinolytic agent that has evidence of reducing blood loss during arthroplasty surgeries. This study aims to evaluate the efficacy and safety of tranexamic acid in patients undergoing hip surgeries.

Materials and methods

This is a cross-sectional study of the patients during the period of May 2020 to April 2021. Forty-eight patients who underwent hip surgery during this period were taken up for the study. Patients were divided into the following groups: group T (n=24) and group P (n=24). Group T received tranexamic acid 10 mg/kg intravenously, as a bolus slowly, 30 minutes prior to skin incision and 1 mg/kg/h intravenous infusion till the closure of skin incision. Group P received normal saline 0.1 ml/kg intravenously, as a bolus slowly, 30 minutes prior to skin incision, and then 1 ml/kg/h intravenous infusion till the skin closure. The primary outcome measured was the total blood loss using Gross and Nadel formulaand the secondary outcomes measured were packed red blood cell (PRBC) transfusion requirement, length of hospital stay, drop-in hematocrit value, ambulation time, and incidence of any other adverse event between the two groups.

Results

The total blood loss in group T patients was 474.12 (± 90.35) ml and in group P was 647.41 (± 114.58) ml, the p-value was <0.001 which was statistically significant. The overall PRBC transfusion rate was 75% (18 patients) in group P and 37.5% (nine patients) in group T with a p-value of 0.020. Nine (37.5%) patients included in group T began to ambulate within 24 hours of surgery while six patients in group P were ambulated within 24 hours with a p-value of <0.001.

Conclusion

Preoperative infusion of tranexamic acid is effective in reducing intraoperative blood loss and blood transfusion requirement rates. It is also safe and efficacious in patients undergoing hip surgeries.

## Introduction

In India, 40,000 total hip arthroplasties are being performed each year [1]. Hip fractures are becoming more common in today's population. Hip fractures are caused by a variety of factors, including falls and decreasing bone mineral density in the elderly. It is estimated that by the year 2050 around 6.3 million estimated hip fractures may occur per year [2]. Management of hip fractures and replacement surgeries might be associated with substantial blood loss which may lead to perioperative anemia. Perioperative blood loss is often managed with blood transfusion, with reported blood transfusion rates ranging from 11-67% [3,4]. Allogenic blood transfusion might increase the risk of disease transmission and transfusion reactions and it might be associated with an increased rate of peri-prosthetic infections [5]. It may also cause a hemolytic reaction, cardiovascular dysfunction, and increased hospitalization. Postoperative mortality rate has been reported as 10% at one month and 30% at one year [6]. Several techniques have been described for the management of blood loss including administration of thromboplastic agents, topical freezing saline, engaging hypotension, and blood transfusion. Recently pharmacological substances such as tranexamic acid is used to minimize blood loss perioperatively.

Tranexamic acid (TXA) is a synthetic amino acid that carries out its effect by its antifibrinolytic action [7]. Fibrin is a protein, arranged in long fibrous chains, which is usually formed from fibrinogen, a protein produced in the liver and present in blood plasma. TXA prevents the degradation of fibrin. It acts by inhibiting the lysine binding site on plasminogen, thereby destroying plasminogen linkage with fibrin to form plasmin, which usually creates the effect of fibrinolysis. By the process of reducing fibrinolysis, it helps in the reduction of blood loss resulting from the trauma due to surgery [8].

Several former studies have suggested that the intravenous route of TXA would minimize blood loss during arthroplasty; however, there are insufficient studies to demonstrate the safety and efficacy of TXA in hip fracture surgeries [9-11]. The current study is conducted to compare the efficacy and safety of intravenous TXA with placebo in patients undergoing surgery for hip fractures.

## Materials and methods

This is a prospective cross-sectional study conducted on patients admitted to the Department of Orthopedics attached to a tertiary hospital in Kolar from May 2020 to April 2021. The Institutional Ethics Committee of Sri Devaraj Urs Medical College approved the study with approval No. DMC/KLR/IEC/51/2021. This study is also submitted to the Clinical Trial Registry of India with the trial acknowledgment number REF/2021/07/045007.

The study includes the patients who are in the age group of 30-90 years of either sex undergoing elective hip surgeries of hemiarthroplasty or hip replacement, fractures involving the neck femur, with normal coagulation report and hemoglobin >9 g/dL. Patients with a previous history of hip surgeries, other associated fractures, pregnancy, history of deep vein thrombosis, or hepatic insufficiency were excluded. Preoperative general and systemic examinations were done in all the patients. Baseline blood investigations like renal function tests, complete blood count, coagulation profile, serology, electrocardiogram, and radiograph of chest were done. All of the hip procedures were performed by top surgeons with more than 15 years of arthroplasty expertise. The patients underwent hemiarthroplasty or total hip replacement following the neck of femur fracture. All the patients underwent replacement with bipolar prosthesis or total hip replacement through Moore’s approach.

After meeting the inclusion and exclusion criteria, a total of 48 patients was included for this study. The patients were divided into two groups on the basis of picking up lots as group T and group P before shifting to the operation theatre (OT). Twenty-four patients in each group were considered for the study.

The patients in group T received TXA at 10 mg/kg intravenously, slowly as a bolus, 30 minutes prior to the skin and then 1 mg/kg/h intravenous till the closure of the incision. Group P patients were from the control group and received normal saline 0.1 ml/kg intravenously, as a bolus slowly, 30 minutes before the incision, and then 1 mg/kg/h till the closure.

On the first postoperative day, repeat complete blood cell count was sent. Packet red blood cell (PRBC) was transfused to those patients whose hemoglobin (Hb) was <10 g/dL. The primary outcomes were total blood loss and the secondary outcomes were PRBC transfusion requirement, length of hospital stay, drop-in hematocrit value, ambulation time, and incidence of any other adverse event. Total blood loss was calculated using the Gross and Nadel formula [12,13].

Total blood loss = patient blood volume (PBV) × (hematocrit {HCT} pre - HCT post) / HCT avg

Here, HCT pre refers to preoperative HCT, HCT post to HCT on the morning of postoperative day three, and HCT avg to the average of HCT pre and HCT post. Patient blood volume (PBV) in liters was calculated using the below formula.

PBV = k1 × height (m)^3^ + k2 × weight (kg) + k3

Here, k1 = 0.3669, k2 = 0.03219, and k3 = 0.6041 for men; and k1 = 0.3561, k2 = 0.03308, and k3 = 0.1833 for women.

Moore’s approach

It is the most common approach used for the exposure of the hip joint. It is popularized by Moore. It is also called the Southern approach. This approach allows easy, quick, and safe access to the joint. They do not interfere with the abductor mechanism of the hip. So, they prevent the loss of abductor power in the immediate postoperative period. It also allows excellent exposure of the femoral shaft; thus, it also helps in joint replacement surgeries.

Statistical analysis

Data were entered using Microsoft Excel and analyzed using the Statistical Package for Social Science (SPSS) standard version 20 (Armonk, NY: IBM Corp.). All continuous variables were summarized using mean (SD) depending on the normality of the distribution. Categorical variables were summarized using proportions. Normality was assessed using the Shapiro-Wilk test and homogeneity of variance was checked using the Levene test. Comparison of categorical variables across study groups was done using the chi-square test. The blood loss (in ml) was reported using mean along with standard error (SE). The comparison of participant characteristics between the tranexamic acid group and controls was done using the independent Student’s t-test and chi-square test.

## Results

During the study period, hemiarthroplasty and total hip replacement were performed on 52 patients. After meeting the inclusion and exclusion criteria, 48 patients were included in the study. Twenty-four patients who received TXA were included in group T and 24 patients who received normal saline were included in group P.

The mean age of the patients in group P is 58.1 years and in group T is 61.1 years. There were 21 males (p-value: 0.251) and 27 females (p-value: 0.383) engaged in the study. Twenty-six patients had neck of left femur fracture while 22 had on the right side. There were 37 patients who underwent bipolar hemiarthroplasty while 11 underwent total hip arthroplasty (p-value: 0.731). The description of the participants is shown in Table 1.

**Table 1:** Description of study participants (n=48). Group P: placebo group; group T: TXA group; TXA: tranexamic acid

Variable		Group P (n=24)	Group T (n=24)
Age (years)		58.1 (9.6)	61.1 (8.5)
Gender	Male	9 (37.5%)	12 (50.0%)
	Female	15 (62.5%)	12 (50.0%)
Side	Neck of femur fracture - left side	12 (50.0%)	14 (58.3%)
	Neck of femur fracture - right side	12 (50.0%)	10 (41.7%)
Type of procedure	Bipolar prosthesis	19 (79.2%)	18 (75.0%)
	Total hip replacement	5 (20.8%)	6 (25.0%)

The total blood loss in group T patients was 474.12 ml (± 90.35) and in group P was 647.41 ml (± 114.58). The statistical analysis was made using independent Student’s t-test and the p-value was <0.001 which was statistically significant. The overall PRBC transfusion rate was 75% (18 patients) in group P and 37.5% (nine patients) in group T with a p-value of 0.020. Out of 18 patients in group P, two patients received two pints of PRBC transfusion. The HCT drop was higher in group P postoperatively (6.27 ± 2.82% in group P vs 5.65 ± 1.89% in group T) with a p-value of 0.369. Nine (37.5%) patients included in group T began to ambulate within 24 hours of surgery while six patients in group P were ambulated within 24 hours with a p-value of <0.001 (Table 2). The complications experienced by the patients are listed out in Table 3.

**Table 2:** Comparison of outcomes between the two groups (n=48). Group P: placebo group; group T: TXA group; TXA: tranexamic acid; HCT: hematocrit

Variable		Group P (n=24)	Group T (n=24)	p-Value
Hematocrit	Preoperative	36.46 (2.89%)	34.69 (3.98)	0.369
	Postoperative	30.18 (3.04%)	29.04 (2.66)	
	Drop in HCT	6.27 (2.82%)	5.65 (1.89)	
Requirement for blood transfusion	No requirement	6 (25%)	15 (62.5%)	0.020
	1 unit	16 (66.7%)	9 (37.5%)	
	2 unit	2 (8.3%)	0	
Blood loss		647.41 ml (114.58)	474.12 ml (90.35)	<0.001
Ambulation time	Less than 24 hours	6 (25.0%)	9 (37.5%)	0.350
	More than 24 hours	18 (75.0%)	15 (92.5%)	
Duration of hospital stay after surgery		9.75 (2.26)	7.20 (1.86)	<0.001

**Table 3:** Complications encountered by the patients. Group P: placebo group; group T: TXA group; TXA: tranexamic acid

Complications	Group P	Group T
No complications	19	20
Deep vein thrombosis	0	1
Pulmonary complications	1	1
Superficial infection	4	2
Implant failure	Nil	Nil

## Discussion

**Figure 1:**
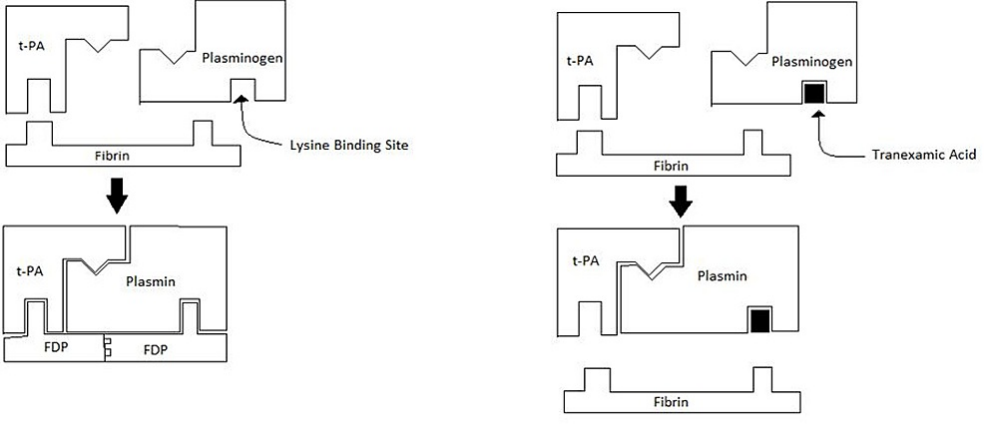
Mechanism of action of tranexamic acid. t-PA: tissue plasminogen activator; FDP: fibrin degradation products

This cohort study concludes that TXA is safe and beneficial for minimizing blood loss, PRBC transfusion rate, length of hospital stay, and ambulation time in patients after hip surgery. The mechanism of action of TXA is given in Figure 1. Elderly patients who are undergoing the procedure are more susceptible to blood loss and complications such as thrombosis when compared to the younger patients, mostly because of their higher comorbidities such as chronic pulmonary disease, congestive heart failure, diabetes, cerebrovascular disease, acute or previous myocardial infarction, peripheral vascular disease, and chronic renal failure. In spite of the additional demands and risks in geriatric hip fractures, they have been quite underrepresented among the many studies.

Some studies suggest that there is an increase in the incidence of deep vein thrombosis, pulmonary embolism, and cerebrovascular diseases in patients who had received TXA intravenously for hemostasis during surgery [14]. In this study, one patient who received TXA during surgery experienced deep vein thrombosis four days after surgery. It was confirmed by venous duplex ultrasonography. Following cardiovascular physician advice, low molecular weight heparin was started for the patient. One of the patients who received TXA became breathless, a spiral CT was recommended suspecting pulmonary embolism. However, no signs of embolism were seen in CT. The patient was given conservative treatment, and the condition gradually improved.

In this study, 37.5% of patients who received TXA intraoperatively were ambulated within 24 hours of surgery while only 25% of patients were mobilized in the placebo group but this was not statistically significant. Various studies done by Zufferey et al., Lee et al., Vijay et al., and Tengberg et al. have proved the role of tranexamic acid in reducing blood loss in patients undergoing hip surgeries [14-17]. Various studies of TXA in hip fracture procedures are shown in Table 4.

**Table 4:** Studies of TXA in hip fracture procedures. RCT: randomized control trial; TXA: tranexamic acid

Study	Design	Sample	Surgery type	TXA regimen	Conclusion
Zufferey et al. [14]	RCT	110	Arthroplasty, dynamic hip screw, intramedullary nail	15 mg/kg prior to surgery and three hours later	TXA is effective but not safe
Lee et al. [15]	Cohort	271	Hemiarthroplasty	1 g bolus preoperative	TXA is safe and cost-effective
Sadeghi et al. [18]	RCT	67	Internal fixation and hemiarthroplasty	15 mg/kg preoperative	TXA significantly reduces blood loss
Baruah et al. [19]	RCT	60	Dynamic hip screw	15 mg/kg preoperative	TXA is safe and effective
Watts et al. [20]	RCT	138	Hemiarthroplasty or total hip replacement	Two doses of 15 mg/kg IV TXA before incision and at wound closure	TXA was safe to reduce blood loss and it reduces the need for transfusion
Emara et al. [21]	RCT	60	Hemiarthroplasty	10 mg/kg prior to surgery and 5 mg/kg/hr infusion till the end of surgery or 1, 0.5 g for topical irrigation	Topical TXA is safer than intravenous TXA
Mohib et al. [22]	RCT	100	-	15 mg/kg preoperative and three hours later	TXA is effective and safe

Different studies have established the efficacy of TXA in reducing blood loss in various approaches to hip surgeries [14-22]. However, the current study is specific only to Moore's approach to hip surgeries. Among all fibrinolytics, TXA has been shown to be superior to aprotinin and epsilon-amino-caproic acid for effectively reducing blood loss, having less incidence of allergic reactions, and less cost of treatment [23].

Various dosages of TXA have been studied with respect to decreased blood loss in hip surgeries. Malhotra et al. showed decreased blood loss when patients were given 15 mg/kg of TXA as a single bolus prior to incision [24]. Yamasaki et al. used higher dose of 20 mg/kg of TXA as a single bolus dose [25]. Benoni et al. administered TXA three hours after surgery but failed to show any significant reduction in blood loss [26]. Horrow et al. showed that larger doses don’t have additional hemostatic benefits [27].

Limitations

This study was conducted only in adult patients who were undergoing elective hip surgeries. The duration of the surgery is not analyzed in the study which may also affect blood loss. Further studies are needed to examine the risks and benefits of TXA use in pediatric age group. It has also been suggested that age may significantly reduce the impact of TXA clearance. The fact that this study was conducted at only one center may also limit the generalizability of the results as the transfusion thresholds differ between centers and even between anesthesiologists.

## Conclusions

According to the findings of this cohort study, TXA administration reduces transfusion rates after hip surgeries, especially when Moore's technique is used. Our results corroborate with prior TXA studies in orthopedics. TXA should be studied further to depict the dosage, method, and time of administration. TXA clearance and the optimal dose regimen for different age groups should be determined. While there was no evidence of a rise in problems, prospective studies should be conducted in the future to track the incidence of adverse events.
